# Tightly integrated single- and multi-crystal data collection strategy calculation and parallelized data processing in *JBluIce* beamline control system

**DOI:** 10.1107/S1600576714022730

**Published:** 2014-11-18

**Authors:** Sudhir Babu Pothineni, Nagarajan Venugopalan, Craig M. Ogata, Mark C. Hilgart, Sergey Stepanov, Ruslan Sanishvili, Michael Becker, Graeme Winter, Nicholas K. Sauter, Janet L. Smith, Robert F. Fischetti

**Affiliations:** a GM/CA@APS, Argonne National Laboratory, 9700 South Cass Avenue, Lemont, IL 60439, USA; b Diamond Light Source, Oxfordshire OX11 0QX, UK; c Lawrence Berkeley National Laboratory, One Cyclotron Road, Berkeley, CA 94720, USA; d Life Sciences Institute and Department of Biological Chemistry, University of Michigan, Ann Arbor, MI 48109, USA

**Keywords:** automated data processing, multi-crystal data collection strategies, X-ray crystallography, *Grid Engine*

## Abstract

The calculation of single- and multi-crystal data collection strategies and a data processing pipeline have been tightly integrated into the macromolecular crystallographic data acquisition and beamline control software *JBluIce*. Both tasks employ wrapper scripts around existing crystallographic software. *JBluIce* executes scripts through a distributed resource management system to make efficient use of all available computing resources through parallel processing. The *JBluIce* single-crystal data collection strategy feature uses a choice of strategy programs to help users rank sample crystals and collect data. The strategy results can be conveniently exported to a data collection run. The *JBluIce* multi-crystal strategy feature calculates a collection strategy to optimize coverage of reciprocal space in cases where incomplete data are available from previous samples. The *JBluIce* data processing runs simultaneously with data collection using a choice of data reduction wrappers for integration and scaling of newly collected data, with an option for merging with pre-existing data. Data are processed separately if collected from multiple sites on a crystal or from multiple crystals, then scaled and merged. Results from all strategy and processing calculations are displayed in relevant tabs of *JBluIce*.

## Abstract 2

Single- and multi-crystal data collection strategy and automated data processing have been tightly integrated into the *JBluIce* graphical user interface. *Grid Engine* is used to distribute these processes into multiple workstations to make efficient use of all available computing resources.

## 1. Introduction   

During the past decade, automation at synchrotron macromolecular crystallography beamlines has developed to a point where the final output is not only a diffraction dataset but also an interpretable electron density map or a structure, which can be available within minutes after data are collected. To achieve this, a good data collection strategy calculation is essential, including specification of the minimum goniometer angular range to record complete data, as well as the maximum angular width of a diffraction image and the detector distance suited to the unit-cell dimensions and diffraction limit of the crystal. For radiation-sensitive crystals from which only incomplete data can be collected, it is also important to sample the largest possible unique volume of reciprocal space in the minimum rotation range. During or after the data collection, key results such as data completeness, data quality, and presence of experimental errors or non-optimal data collection parameters must be fed back rapidly so the experimenter can evaluate the success of the data collection or suitability of the protocol. This is possible only if the data are processed and the quality parameters are monitored in parallel with data collection, in particular while the crystal is still mounted on the goniometer.

After a crystal is mounted and centered, users generally record and inspect test diffraction images. The common practice is to record either one image, two orthogonal images or a small wedge of data and to index them using software such as *HKL-2000* (Otwinowski & Minor, 1997 ▶), *MOSFLM*/*iMOSFLM* (Leslie, 1999 ▶) or *XDS* (Kabsch, 2010 ▶). On the basis of the output of these programs and in some cases an additional strategy calculation, users enter data collection parameters into the beamline control software. This is in general a time-consuming process and is not an efficient use of valuable synchrotron beam time. Therefore, rapid and reliable interfaces such as *WebIce* (González *et al.*, 2008 ▶) and *EDNA* (Incardona *et al.*, 2009 ▶) have been integrated into the data acquisition software at many beamlines to provide automated indexing, cell refinement and strategy calculation. The results are displayed either in the beamline control software or through an external interface. For example, the SSRL *Blu-Ice* data acquisition software (McPhillips *et al.*, 2002 ▶) includes integration with *WebIce*; software from NE-CAT at the Advanced Photon Source provides *RAPD*, a web-based strategy and data processing interface integrated with the beamline controls (Murphy *et al.*, 2011 ▶); and *EDNA* is integrated with both *OpenGDA* data acquisition at the Diamond Light Source (OpenGDA, 2010 ▶) and *MxCube* data acquisition at the ESRF (Gabadinho *et al.*, 2010 ▶). In addition, several packages for automated data processing have been developed, for example *xia2* (Winter, 2010 ▶), *fast_dp* (Winter & McAuley, 2011 ▶), *autoPROC* (Vonrhein *et al.*, 2011 ▶), *AutoDrug* (Tsai *et al.*, 2013 ▶) and a local ESRF package (Monaco *et al.*, 2013 ▶). These packages wrap around *XDS* (Kabsch, 2010 ▶) and exploit its use of multiple CPU cores for fast parallel data processing with minimal human intervention, thus enabling their integration into the beamline user interface for use in real time. They also pipe the *XDS* output into the *CCP4 *suite (Collaborative Computational Project, Number 4, 1994 ▶), where they direct the assessment of screw axes, further scaling and merging, and reduction of reflection intensities to structure amplitudes.

At the General Medicine and Cancer Institutes beamlines at the Advanced Photon Source (GM/CA@APS), *JBluIce*, a unified graphical frontend to the beamline control and data acquisition system (Stepanov *et al.*, 2011 ▶), provides an integrated and uniform user interface for all aspects of beamline operation, crystal screening and data collection. In this paper, we provide details of the *JBluIce* implementation of an automated data collection strategy calculation. As a next step, we have supplied *JBluIce* with a multi-crystal strategy pipeline to generate a strategy for the current crystal based on processed data from previous crystals of the same type. This is an important addition as the development of microcrystallography and fast detectors and the complication of radiation damage have reintroduced multi-crystal data collection. Implementation of the multi-crystal strategy calculation has also been undertaken in *STAC* (Brockhauser *et al.*, 2013 ▶) using the *STRATEGY* software (Ravelli *et al.*, 1997 ▶). We further describe the implementation of an automated data processing pipeline suitable for single-crystal and multi-crystal datasets.

## 2. *JBluIce*, *Grid Engine* and *WebIce*   

The GM/CA@APS beamline control software *JBluIce-EPICS* (Stepanov *et al.*, 2011 ▶) was developed with Java Eclipse RCP for the *JBluIce* graphical user interface (GUI) design and *EPICS* for distributed hardware control. *JBluIce* maintains the look and feel of *Blu-Ice*, the SSRL beamline control software (McPhillips *et al.*, 2002 ▶), while providing flexibility to add new features and deploy advanced hardware capabilities through its object-oriented multi-threaded and model view controller (MVC) architecture. The pure-Java-based GUI of *JBluIce* is the single control point for data collection at Sector 23 beamlines at the APS. The complex array of tasks available to the experimenter is organized under tabs (Hutch, Sample, Screening, Raster, Scan, Collect, Analysis, Users and Log) of the *JBluIce* GUI.

To distribute the tasks of strategy calculation and data processing, *JBluIce* uses the SUN/Oracle *Grid Engine* (http://gridscheduler.sourceforge.net), an open-source distributed resource management system that monitors the CPU and memory usage of all available beamline computers connected to a shared storage array and distributes jobs among them. *JBluIce* submits jobs to a *sgemaster* daemon that runs on a *Grid Engine* master node and schedules jobs on execution nodes where they are managed by a *sge_execd* daemon. The interface between *JBluIce* and *sgemaster* is based on Java bindings to the distributed resource management application API (DRMAA; Troger *et al.*, 2007 ▶). *Grid Engine* was selected for its high scalability, cost effectiveness, ease of maintenance and high throughput. It helps to optimize the use of several multi-core single- or dual-CPU workstations at the beamlines. In the GM/CA environment, experimenters typically use between two and six workstations among the ten available. *Grid Engine* speeds data processing by automatically and flexibly scheduling jobs to the workstations with the least-loaded CPUs.

At the GM/CA@APS beamline computing system, user home directories are arranged on a shared storage array and made available to all workstations, which are connected *via* a global file system (GFS, Red Hat). User authentication is based on lightweight directory access protocol (LDAP). *Grid Engine* runs as a root user and changes its effective user-id to the user-id of the submitted job; thus no separate authentication is needed to execute strategy and data processing tasks from *JBluIce*.

For the high-level tasks of indexing and strategy calculation, we stripped the standalone web server frontend from *WebIce* and ported the core scripts to be called directly from *JBluIce*. These include scripts for the programs *LABELIT* (Sauter *et al.*, 2004 ▶) for auto-indexing, *MOSFLM* for integration, and either *BEST* (Popov & Bourenkov, 2003 ▶) or *MOSFLM* for strategy calculation. Modifications of *WebIce* to operate within *JBluIce* include the introduction of *Grid Engine* for faster execution of indexing and strategy scripts, the addition of a MySQL database for storing intermediate results, and the display of all results within a *JBluIce* window rather than a web browser. We retained the *WebIce* image server, which generates jpeg snapshots of diffraction images, and the core C-shell scripts for execution of *LABELIT*, *MOSFLM* and *BEST*. The SSRL *WebIce* and *JBluIce* implementations are compared in Table 1 ▶.

In addition to the *WebIce*-based crystal evaluation and strategy calculation, *Grid Engine* is also used for two data processing pipelines, *fast_dp* (Winter & McAuley, 2011 ▶) and *GMCAproc* (developed in-house), which are wrappers around *XDS*, *POINTLESS* (Evans, 2006 ▶), *AIMLESS* (Evans, 2011 ▶), *SCALA* (Evans, 2006 ▶) and *TRUNCATE* (French & Wilson, 1978 ▶).

## 3. Sample handling and screening   

**Figure 1:**
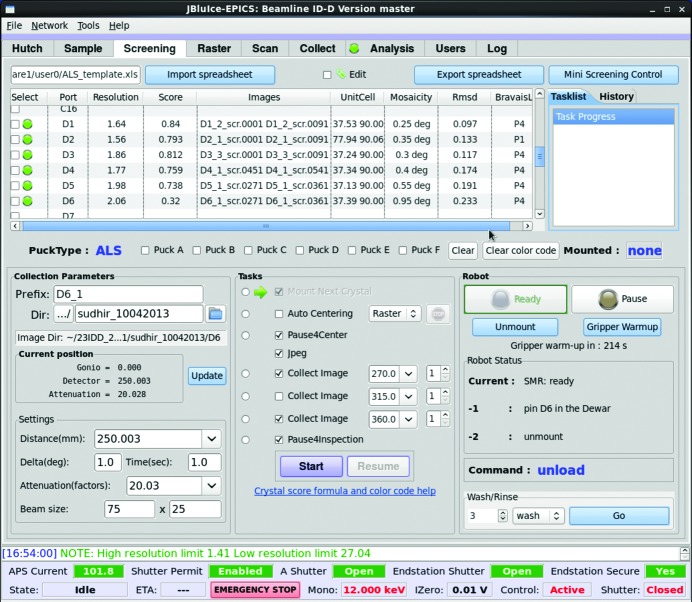
Screening tab of *JBluIce*. Sample information is shown as a table containing the indexing results and a quality score for each sample. Sample-specific information (port, file prefix, directory, comment and protein information) are imported from the uploaded spreadsheet (not shown in the figure), while indexing results such as unit cell, mosaicity, r.m.s.d., score, resolution, Bravais lattice and images used are displayed in the spreadsheet. The user can set up parameters for collection of test images and other tasks. The current status of the robot is also shown.

The data pipeline begins with sample screening. Nearly all users have cryo-cooled samples in pucks that are loaded into an automounter dewar (Makarov *et al.*, 2007 ▶, 2011 ▶). Sample-specific information is pre-loaded into a formatted spreadsheet, which is imported into the *JBluIce* Screening tab (Fig. 1 ▶). Users select samples for screening as well as a set of operations for each sample, for example to mount, to auto-center optically (Pothineni *et al.*, 2006 ▶) or with diffraction (Raster) (Hilgart *et al.*, 2011 ▶), and to collect test diffraction images at up to three goniometer orientations. The crystal identification and directory information from the spreadsheet are exported automatically to the *JBluIce* Collect, Scan and Raster tabs. Data collection parameters such as detector distance, image angular width, exposure time, beam size and attenuation factor can be selected in the Screening tab for the test images.

After collecting at least two images from a sample in the Screening tab, *JBluIce* initiates auto-indexing with *LABELIT* and integration with *MOSFLM*. The resulting crystal information including apparent space group, unit-cell parameters, resolution limit and an estimate of mosaicity [on the presumption that the breadth of Bragg peaks is due entirely to sample mosaicity, which is the case on GMCA beamlines with beam divergence of <0.01° (Fischetti *et al.*, 2009 ▶)] are displayed in the Screening tab spreadsheet, along with an empirical *WebIce* quality score. Users can compare the results from screened samples and then select samples for data collection.

## 4. Single-crystal data collection strategy   

**Figure 2:**
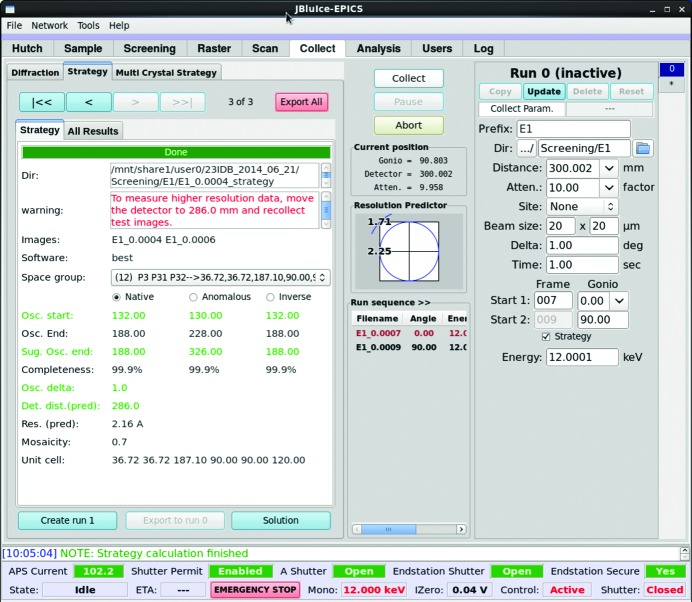
*JBluIce* Collect tab with Strategy sub-tab selected (left side of window). The parameters listed are working directory (Dir), warning, list of possible Laue groups in a drop-down menu (Space group), starting (Osc. start) and ending (Osc. End) goniometer angles for collection of data to achieve maximum completeness (Completeness), suggested oscillation end (Sug. Osc. end), image angular width (Osc. delta), optimal detector distance [Det. dist.(pred)] for X-ray energy and predicted resolution limit [Res. (pred)], apparent sample mosaicity, and unit-cell constants. The collection parameters for the test images, such as detector distance, angular width, exposure time *etc*., are shown on the right side of the figure.

A strategy calculation using either *BEST* or *MOSFLM* is initiated whenever the Screening tab successfully indexes and integrates two images, and results are displayed directly in the *JBluIce* Strategy sub-tab of the Collect tab (Fig. 2 ▶). In the case of manually mounted crystals or sites identified in the Raster tab, the strategy option is available in the ‘0’ run tab of the Collect tab.

**Figure 3:**
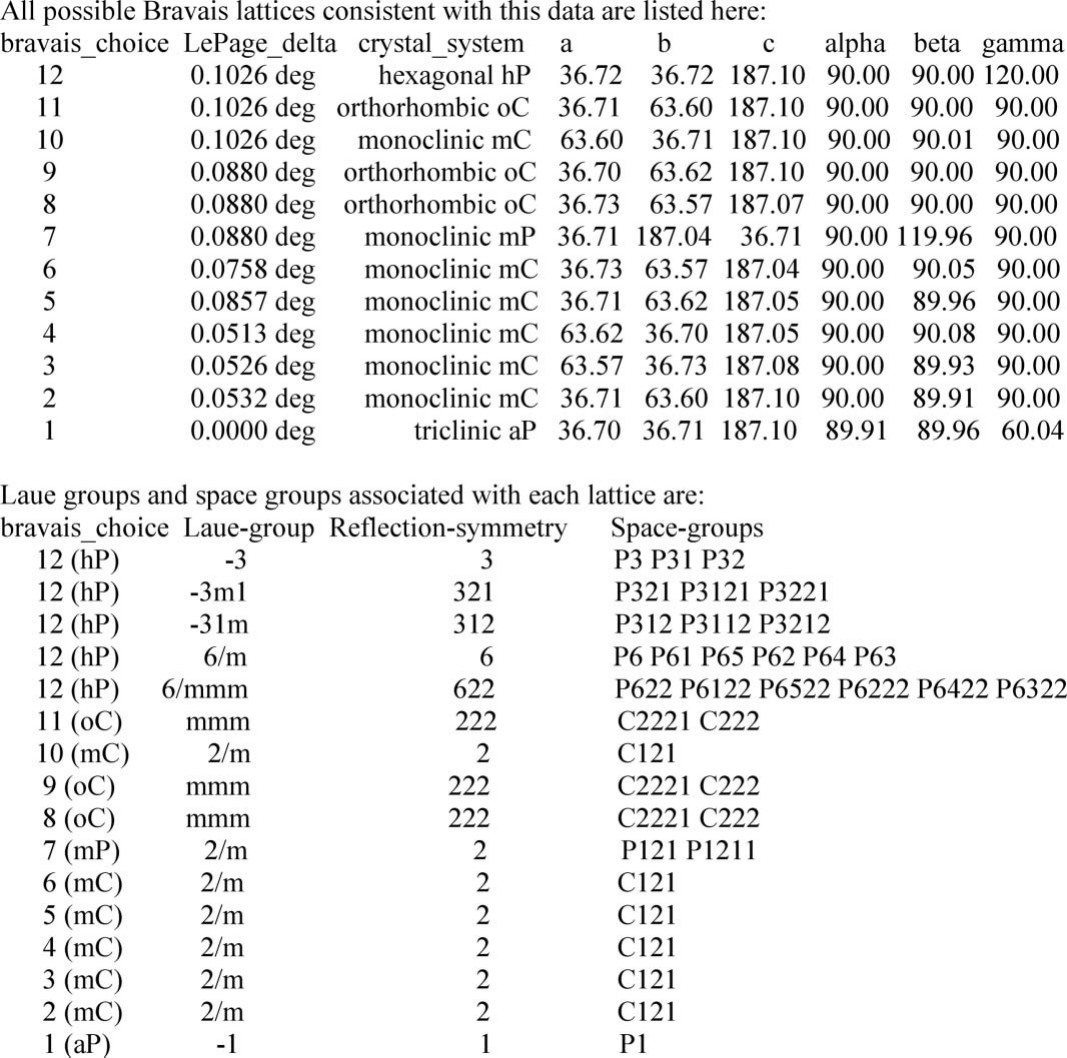
Example *LABELIT* Laue choices log file. *JBluIce* can produce a strategy for any of the 12 solutions. In this example, strategies for Laue groups 3, 3*m*1, 31*m*, 6/*m* and 6/*mmm* are calculated in parallel. The other solutions can be selected for strategy calculation from the pull-down menu on the Strategy sub-tab.

A pull-down space-group menu displays indexing solutions, which have been parsed and filtered from the output of the labelit.laue_choices command (example in Fig. 3 ▶). Solutions belonging to the same crystal_system and with very similar unit-cell axes are filtered out, and only one among the similar solutions is shown in the pull-down menu of the *JBluIce* Strategy sub-tab. All *LABELIT* solutions are available by clicking the ‘Solution’ button. By default, strategies for each of the highest-symmetry Laue groups identified by *LABELIT* are calculated in parallel through *Grid Engine*. In the Fig. 3 ▶ example, this includes the three trigonal and two hexagonal Laue groups. Users can choose a different indexing solution from the list of space groups, which will initiate a new strategy calculation. They also can select between *BEST* or *MOSFLM* for strategy calculations through the *JBluIce* Options menu (under the Tools pull-down menu in Fig. 2 ▶).

A typical data collection strategy provides suggested starting and ending goniometer angles, image angular width (Osc. delta), detector distance and estimated data completeness. Other parameters such as the apparent space group, unit-cell constants, estimated crystal mosaicity and predicted resolution limit are also displayed in both the Strategy sub-tab (Fig. 2 ▶) and the Screening tab (Fig. 1 ▶). The results are displayed for native data and two anomalous data collection options, namely, anomalous continuous and anomalous inverse (for true Friedel pairs). The anomalous-continuous option suggests twice the minimum continuous angular range necessary for anomalous coverage, in order to provide redundancy similar to the anomalous-inverse option. Strategy results can be exported to a collect run (‘Create run #’ button) for collecting data on the given crystal.

The Strategy sub-tab has a field for displaying errors/warnings from *LABELIT*, *MOSFLM* or *BEST*, such as strategy calculation failures and infeasible experimental conditions. The warning circumstances and messages are designed on the basis of our experience of a number of actual scenarios that include warnings of ‘non-zero two-theta: strategy not supported’ when the detector 2θ is not equal to zero, ‘LABELIT suggests pseudo translation: check LABELIT solution’ when *LABELIT* warns about pseudo translation (Sauter & Zwart, 2009 ▶), ‘no indexing solution’ when *LABELIT* is unable to determine three basis vectors, *etc*. An expert user has the option to modify the generated input files and re-run the strategy as a command line script.

## 5. Multi-crystal strategy   

**Figure 4:**
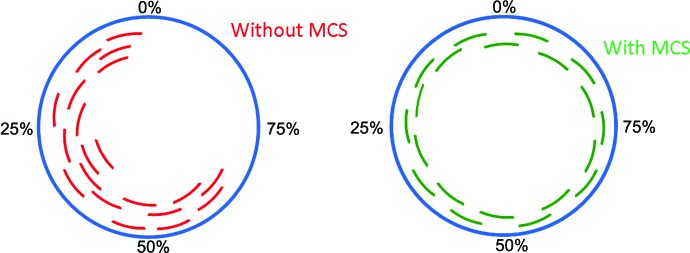
Schematic illustration of data completeness by merger of incomplete data from multiple crystals with and without MCS. An incomplete dataset from each crystal is represented by an arc (green arcs for an example with MCS and red arcs without MCS). A full circle (blue) represents the complete dataset.

The widespread adoption of microcrystallography (Smith *et al.*, 2012 ▶) and the recent demonstration of the benefits of high-multiplicity datasets (Liu *et al.*, 2013 ▶; Diederichs & Karplus, 2013 ▶; Akey *et al.*, 2014 ▶) are vastly increasing the acquisition of multi-crystal datasets. It is now common practice for G Protein-coupled receptor investigators to collect small random wedges of data from multiple microcrystals and to merge the incomplete datasets (Cherezov *et al.*, 2007 ▶, 2009 ▶; Rasmussen *et al.*, 2011 ▶). However, random incomplete datasets can lead to missing wedges of reciprocal space and may reflect inefficient use of beam time (Fig. 4 ▶, left).

**Figure 5:**
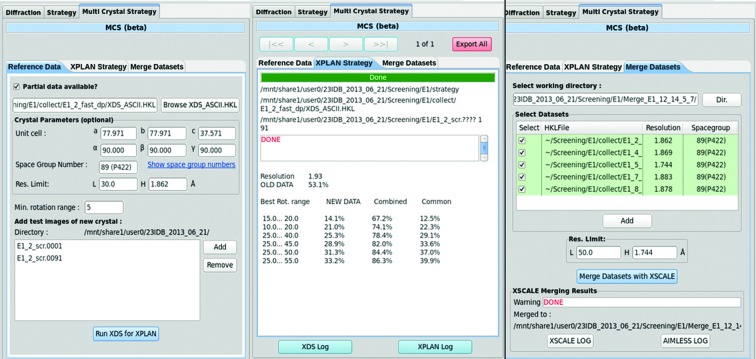
Inner-tabs of the MCS sub-tab in *JBluIce*. The loaded XDS_ASCII.HKL file is shown in the Reference Data tab, with corresponding cell parameters and space group. Test images from the current crystal are also listed in this tab. A multi-crystal strategy for maximum completeness for a new crystal, when combined with existing data, is shown in the XPLAN Strategy tab (‘common’ represents reflections common to new and old data as a percent of all measured data). The Merge Datasets tab can control datasets to include in scaling and resolution limits. The sample is thaumatin; each subset shown in the figure is a small wedge of data collected with a Rayonix MAR300 CCD (distance 300 mm, 1° angular width, 1 s exposure time) and processed in a primitive lattice in Laue group 4/*mmm*.

A multi-crystal strategy (MCS) feature was introduced in *JBluIce* for systematic data collection from multiple crystals, including a strategy for the current crystal based on its orientation and the processed data from earlier crystals. MCS is presented to users as a sub-tab of the *JBluIce* Collect tab, and its pipeline is controlled through three inner-tabs, Reference Data, XPLAN Strategy and Merge Datasets (Fig. 5 ▶).

(*a*) The Reference Data inner-tab is used to load previous data and test images for the current crystal. The previous data can be either from one crystal (XDS_ASCII.HKL file from *XDS* or .sca file from *HKL-2000*) or from several crystals [from *XSCALE* (Kabsch, 2010 ▶) output, also in the form of XDS_ASCII.HKL available from the Merge Datasets inner-tab]. The test diffraction images from the current crystal can be loaded manually or automatically from the Screening tab into this inner-tab. Clicking the ‘Run XDS for XPLAN’ button initiates a strategy calculation.

(*b*) Strategies are calculated to generate maximum completeness from the current crystal for data wedges sized in multiples of a user-specified minimum rotation range (5° in the example shown in Fig. 5 ▶), and the results are displayed on the XPLAN Strategy inner-tab. In the current implementation, the user enters the data collection range from the displayed table to collect new data.

(*c*) The Merge Datasets inner-tab is used to combine reference data with newly collected and processed data using *XSCALE*. The resulting combined unmerged *hkl* data (XDS_ASCII.HKL) can then be used again in the Reference Data inner-tab.

The current implementation does not automatically account for variations in crystal quality, resolution limit and crystal non-isomorphism. To avoid these problems, the user is provided with an option to select/de-select a particular dataset in the Merge Datasets inner-tab.

## 6. Data processing pipeline for several data collection modes in *JBluIce*   

**Figure 6:**
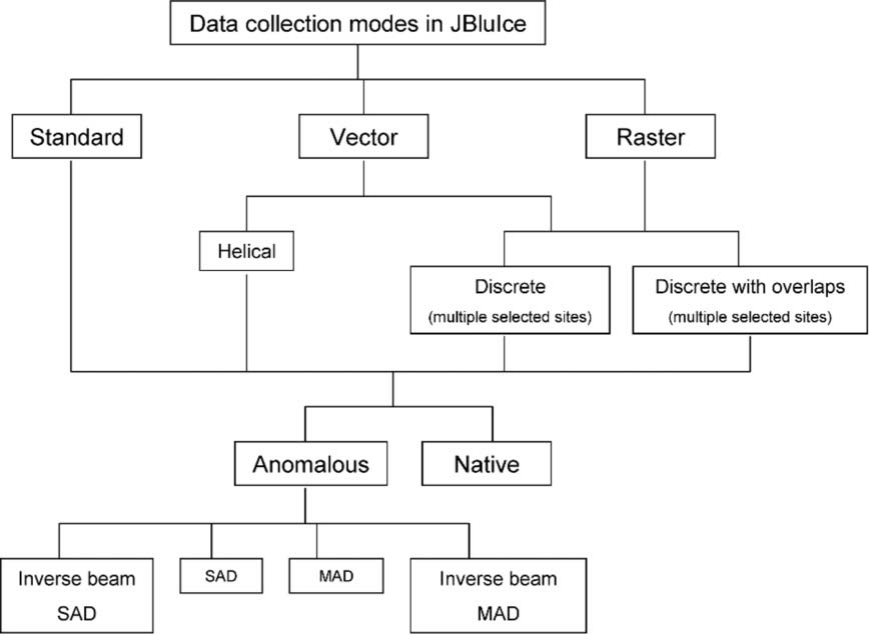
Hierarchy of data collection modes in *JBluIce*.

*JBluIce* has several data collection modes (Hilgart *et al.*, 2011 ▶) to address the varied requirements of the challenging problems users bring to GM/CA, including membrane-protein crystals, frequently grown in lipidic cubic phase, crystals of macromolecular complexes and crystals with large unit cells, which are common with large complexes. The data collection modes are named Standard, Vector and Raster (Fig. 6 ▶). Standard mode has a Native option and an Anomalous option with a choice of single- or multi-wavelength collection. In Vector mode, an angular sweep of data can be partitioned among discrete sites along a crystal or recorded in a continuous helical geometry, with the additional option for inverse-beam geometry with true Friedel pairs collected from the same site in the crystal. Also, Vector-mode data can be collected with angular overlaps between discrete sites to help with scaling the separate sites. Raster mode is for data collection from multiple sites, no matter whether these sites are from a single crystal or from multiple crystals within a single sample loop. The different data collection modes present interesting challenges to the two data processing pipelines, *fast_dp* and *GMCAproc*.

### 6.1. Data processing   

**Figure 7:**
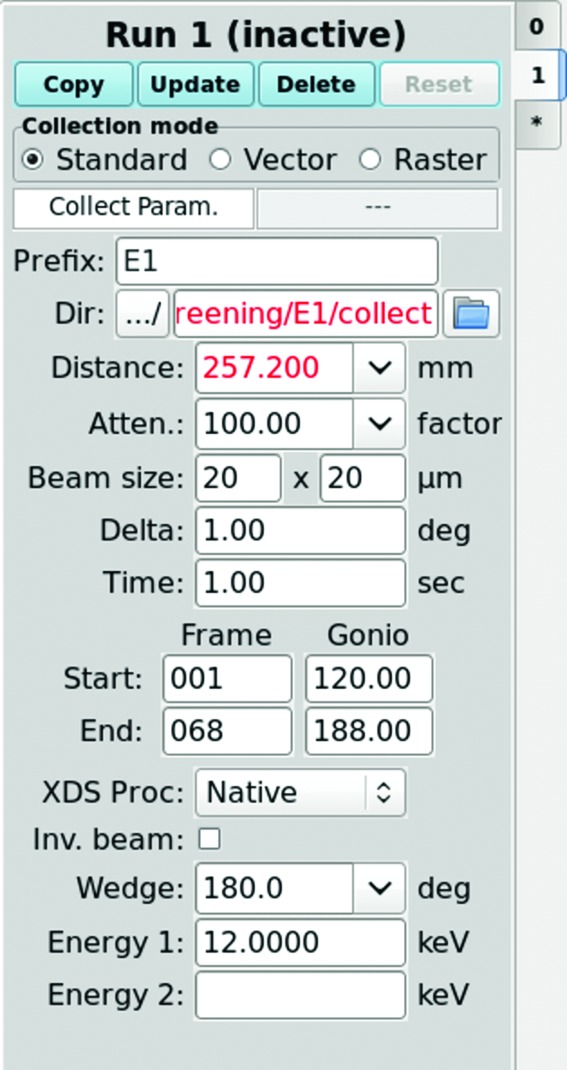
Data collection parameters in the Collect tab of *JBluIce*. The XDS Proc pull-down menu is used to initiate data processing for the specified data collection mode.

Automated data processing is integrated into *JBluIce* for all data collection modes using either the *fast_dp* or *GMCAproc* data-processing pipeline. On the *JBluIce* Collect tab under the ‘XDS Proc’ pull-down menu, users choose the Native or Anomalous option for data processing, or the NONE option for no processing (Fig. 7 ▶). If Native or Anomalous is selected, *XDS* processing is initiated through *Grid Engine* as soon as a given sweep of data is collected (generally 10°, based on our analysis of a large number of user cases). While *JBluIce* continues to collect data, it re-initiates data processing from the first image as each new sweep of data is collected. This process is repeated until all data have been processed for a given collection. Thus the user has updated information about data quality during the collection. Such processed data can be exported for multi-crystal strategy calculation.

**Figure 8:**
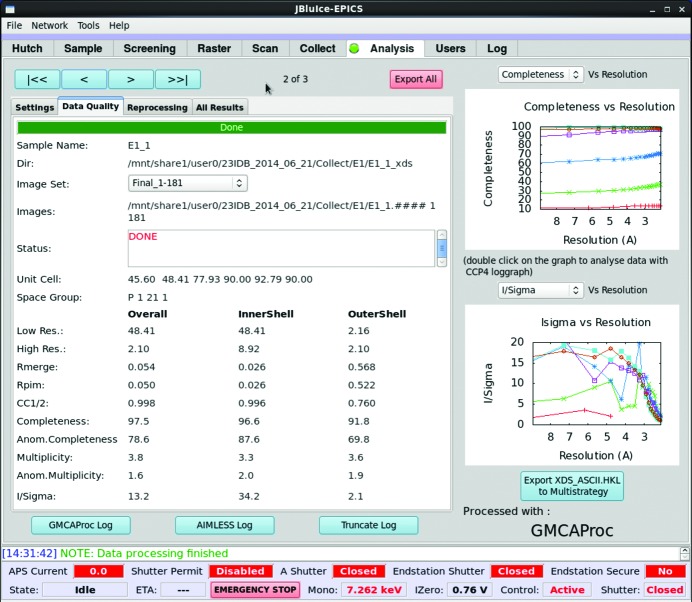
*JBluIce* Analysis tab, showing data quality parameters for a set of images. The statistics shown are from unpublished data collected with a Rayonix MAR300 CCD detector (distance 300 mm, 1° angular width, 1 s exposure time. The data were processed with the pipeline *GMCAproc*; the data quality parameters shown as a table are the parsed output of the program *AIMLESS*. The plots on the right show the quality of data; each colored trace on the graph corresponds to an image subset available in the ‘Image Set’ pull-down menu.

Data quality parameters, such as diffraction limit, completeness, *I*/σ_*I*_, *R*_merge_, *R*_pim_ (Weiss, 2001 ▶) and CC_1/2_ (Karplus & Diederichs, 2012 ▶) for the overall data, and the highest- and lowest-resolution shells, are tabulated in the *JBluIce* Analysis tab (Fig. 8 ▶). The Analysis tab also displays two plots of data quality *versus d* spacing, with three additional plots available *via* a pull-down menu above each plot. These include *R*_merge_, *I*/σ_*I*_, *R*_pim_, completeness and multiplicity. In addition, a button click produces the *XDS*, *AIMLESS* and *TRUNCATE* log files, parsed by *BAUBLES* (Briggs & Cowtan, 2007 ▶) and displayed in a web browser. All relevant data processing files are available in a subfolder of the data collection directory. Scaled data are output in *CCP4* mtz format for convenient use in structure solution.

The two pipelines, *fast_dp* and *GMCAproc*, which are user-selectable from the *JBluIce* options menu, exploit the parallel architecture of *XDS* for indexing and integration, *POINTLESS* for space-group selection, and *AIMLESS* for merging and statistical output. *GMCAproc* uses *TRUNCATE* to generate structure factor amplitudes. *GMCAproc* and *fast_dp* differ in their *XDS* input parameters for auto-indexing and Laue group determination. *fast_dp* employs an internal algorithm for decision making at each stage of *XDS* processing (indexing, integration, scaling), runs multiple CORRECT passes, and integrates only once. *GMCAproc* employs two passes for *XDS*. In the first pass, all *XDS* subroutines (parameter JOB = ALL in the XDS.INP) are run by auto-indexing with 50% of the total diffraction images, integrating in space group *P*1, and performing steps of scaling, Laue group determination and unit-cell refinement in the CORRECT step. In the second pass, the output file GXPARAM.XDS from the CORRECT step of the first pass is replaced as XPARAM.XDS, and *XDS* is run with the parameter JOB = DEFPIX INTEGRATE CORRECT, wherein re-integration is carried out in the correct Laue group (Diederichs, 2008 ▶). *POINTLESS* is used for space-group determination at this stage with the keyword ‘SETTING SYMMETRY-BASED’, followed by *AIMLESS* for statistical output and data merging (keyword ‘scales constant’).

At GM/CA, *fast_dp* is used extensively for data collected in Standard mode, and in Vector mode without overlaps. *GMCAproc*, which functions for data from all collection modes, modifies the *XDS* processing parameters according to the mode. The most important of these is to preserve the crystal orientation matrix for different sweeps of data. Therefore *GMCAproc* is used for data collected in inverse-beam geometry or in Vector mode where sites have small angular overlaps. When multiple sweeps of data are collected from the same crystal, the first sweep of processed data acts as the REFERENCE_DATA_SET for the remaining sweeps. The orientation matrix, space group and unit-cell constants from the first sweep are added to the *XDS* input file (XDS.INP) for the remaining sweeps to maintain a uniform indexing for all sweeps. In the case of data collected from multiple sites in Raster mode, data from each site are processed independently, combined using *POINTLESS*, and merged and scaled using *AIMLESS*.

In addition to automated data processing, a manual reprocessing option is also available within *JBluIce* in the form of a Reprocessing sub-tab of the Analysis tab. The Reprocessing sub-tab gives users the ability to force *XDS* to process data with a specified space group and cell parameters by either entering the values manually or importing them from an earlier strategy calculation. An option to start the *xdsGUI* from *JBluIce* is also available in this sub-tab.

All processing results and metadata (data collection mode, pipeline used, processing directory name, warnings if any *etc*.) from screening, strategy calculation and data processing for a given user account are tabulated in a single MySQL database and displayed in the ‘All Results’ sub-tabs in the corresponding *JBluIce* tabs. These tables can be exported as *Excel* spreadsheets for comparison of results from samples within a project.

### 6.2. Results and discussion   

At GM/CA we chose a grid computing approach by making use of the existing beamline workstations instead of setting up a dedicated computing cluster. The advantage of such an approach is a more efficient use of the computing resources as the workstations are used not only for automated strategy calculation and data processing but simultaneously for all other user computing needs. Each beamline is equipped with ten Intel Xeon multi-core workstations (12–24 cores per workstation). These workstations are used for the automated processes described above and also to collect data, to process data manually, and to run programs to solve and refine structures. Occasionally, some workstations are used by previous users for manual data processing.

To speed up data processing with *XDS*, hyper-threading was enabled, effectively doubling the number of cores on each workstation. The *forkcolspot* and *forkintegrate* scripts of *XDS* were modified to work with *Grid Engine* so that multiple sub-jobs from a single *XDS* job could be spawned to multiple computers. This cluster-based parallelized version of *XDS*, named *xds_par*, is used to process data in a sub-directory of the data collection folder. The parallelization is controlled *via* the *XDS* parameters specifying the maximum number of jobs run in parallel and the maximum number of processors (Diederichs, 2011 ▶).

Several datasets were processed to test the pipelines. A representative dataset from the Pilatus3 6M (900 images, each of 0.2° angular width and 0.2 s exposure time) is shown in Table 2 ▶ for both *fast_dp* and *GMCAproc*, displaying comparable results from the two pipelines. Even though different total times were taken to process the same data with *fast_dp* and *GMCAproc*, the integration times for the two pipelines were generally comparable. The additional time for *GMCAproc* processing was due to the use of 50% of the data for auto-indexing and a second integration step using the correct Laue group (as explained in §6.1). In the case of *fast_dp*, auto-indexing is performed on small wedges of data at the start, middle and end of the dataset, the CORRECT step is run multiple times, and the integration step is run once.

In another benchmark, the *fast_dp* pipeline was run simultaneously with data collection on the Pilatus3 6M detector (Table 3 ▶). Intermediate processing results were available at three points during the data collection, and fully processed data were available 70 s after the data collection completed.

## 7. Conclusions   

Automated data collection strategy calculation and data processing have been tightly integrated into *JBluIce*. The availability of results from strategy calculation and data processing within the graphical interface for beamline and experiment control helps users make informed real-time decisions for a given crystal while it is mounted on the goniometer. This helps both novice and experienced users in the effective use of valuable samples and synchrotron beam time and also in achieving high throughput. With the use of *Grid Engine* we were able to make efficient use of the existing beamline computing resources to achieve real-time data processing.

Although the data acquisition software in general suggests optimized strategy parameters for native and anomalous data collection, our implementation of data processing gives users an option to intervene at each step and to alter the parameters on the basis of their decisions and the specifics of their project and crystal. On-the-fly data processing helps users decide whether data collection achieved the required completeness, resolution and multiplicity. If data are incomplete, users can collect more data from different part of the same crystal or augment with data from additional crystals.

Future plans include optimizing the processing and reprocessing of data from a high-speed Pilatus3 6M detector operating at 100 Hz and extending the strategy feature with calculations of recommended values for exposure time, attenuation and the Garman limit (Owen *et al.*, 2006 ▶) of radiation dose. These improvements will incorporate on-the-fly flux calculations from ‘active’ beamstop measurements (Xu *et al.*, 2010 ▶) combined with calculations of radiation dose with *RADDOSE* (Paithankar *et al.*, 2009 ▶). The architecture of *JBluIce* will facilitate integration of these advanced features with minimal effort.

## Figures and Tables

**Table 1:** Comparison of strategy implementation in *WebIce* and *JBluIce*

	*WebIce*	*JBluIce*
Execution	Crystal-analysis software called by impersonation daemon	*Grid Engine* routing to crystal-analysis software
Intermediate data	Sample information server	MySQL database
Image display	*WebIce* image server	*WebIce* image server
Results display	External web interface	Java interface internal to beamline control software
Strategy calculations	SSRL C-shell scripts	Modified SSRL C-shell scripts
Authentication	Internal server	LDAP based

**Table 2:** Processing results (*AIMLESS* log summary) of unpublished user data using the *fast_dp* and *GMCAproc* pipelines Shutterless data (900 fine-sliced images, 0.2 angular width, 0.2s exposure time) were collected with the Pilatus3 6M detector at beamline 23ID-D.

	*fast_dp*	*GMCAproc*
Time taken (s)	123	173
Space group	*P*321	*P*3_2_21
Unit cell	58.90 58.90 481.45 90.00 90.00 120.00	58.91 58.91 481.10 90.00 90.00 120.00
Low-resolution limit ()	29.97	29.45
High-resolution limit ()	2.57	2.56
*R*_merge_ (within *I*+/*I*)	0.128	0.116
*R*_merge_ (all *I*+ and *I*)	0.135	0.121
*R*_meas_ (within *I*+/*I*)	0.142	0.130
*R*_meas_ (all *I*+ and *I*)	0.143	0.129
*R*_pim_ (within *I*+/*I*)	0.063	0.057
*R*_pim_ (all *I*+ and *I*)	0.047	0.042
*R*_merge_ in top intensity bin	0.056	0.055
Total number of observations	301 302	309 714
Total number unique	32 501	32 778
Mean(*I*/_*I*_)	13.1	14.4
Mean(*I*) half-set correlation CC_1/2_	0.996	0.997
Completeness (%)	99.2	99.5
Multiplicity	9.3	9.4
Anomalous completeness (%)	98.8	99.1
Anomalous multiplicity	5.0	5.1

**Table 3:** *fast_dp* data processing times in parallel with data collection for a thaumatin dataset collected with the Pilatus3 6M detector at beamline 23ID-D

Diffraction data	900 images
Exposure time	0.2 s
Image angular width	0.2
Time for data collection	209 s
1st processing results, images 169	74 s
2nd processing results, images 1319	141 s
3rd processing results, images 1639	213 s
Final processing results, images 1900	279 s
Time for final processing results in *JBluIce* after data collection ends	70 s
